# Autophagy is involved in the protective effect of p21 on LPS-induced cardiac dysfunction

**DOI:** 10.1038/s41419-020-02765-7

**Published:** 2020-07-21

**Authors:** Sihui Huang, Man Xu, Libo Liu, Jingjing Yang, Huibo Wang, Chunxia Wan, Wei Deng, Qizhu Tang

**Affiliations:** 1 Department of Cardiology, Renmin Hospital of Wuhan University, 430060 Wuhan, PR China; 2 Hubei Key Laboratory of Metabolic and Chronic Diseases, 430060 Wuhan, PR China

**Keywords:** Autophagy, Cardiovascular diseases

## Abstract

p21 has emerged as an important protein involved in cardiovascular diseases, but its role remains controversial. Recently, p21 has been reported to mediate inflammatory responses. As inflammatory responses are a feature of sepsis, our study investigated whether p21 has a role in cardiac dysfunction induced by sepsis and analyzed the mechanisms involved. To establish a mouse sepsis model, p21 global knockout (p21KO) and C57BL/6J wild-type (WT) male mice were treated with 5 mg/kg LPS intraperitoneally for 6, 24, or 48 h. After LPS stimulation, the level of p21 had significantly increased in the WT mice and in cardiomyocytes. Cardiac dysfunction induced by LPS was markedly aggravated in p21KO mice relative to that of WT mice. Downregulation of p21 expression exacerbated the LPS-mediated inflammatory response, and it increased oxidative stress as well as mitochondrial damage in the heart and in cardiomyocytes. In contrast, overexpressing p21 attenuated the increase of TNFα and promoted the increase of SOD2. Moreover, p21 regulated the LPS-induced autophagy activation; that is, the increase in autophagy was impaired when p21 expression was decreased, whereas the increase was significant when p21 was overexpressed. The autophagy inducer rapamycin partially rescued the cardiac deterioration caused by p21 downregulation in the LPS-stimulated groups. In addition, p21 regulated the autophagy level by interacting with LC3B. These results revealed that p21 controls LPS-induced cardiac dysfunction by modulating inflammatory and oxidative stress, and it is partially dependent on regulating the autophagy level. This study is the first to show that p21 could interact with LC3B to promote autophagy for the improvement of cardiac function during sepsis.

## Introduction

Sepsis is the most common systemic inflammatory response in the clinic. It is mainly caused by the release of large amounts of endotoxins from Gram-negative bacteria into the blood. The active chemical component of endotoxin-induced disease is mainly lipopolysaccharides (LPS)^1^. If not treated promptly, it will proceed to multiple organ failure, and the heart is one of the main organs affected during sepsis. Some studies have shown that sepsis-induced heart dysfunction can be as high as 70%^2^. If there are cardiac complications, the patient’s condition deteriorates rapidly, and the mortality rate will be as high as 50–60%^2^.

These data suggest that cardiac damage plays an important role in septic patients’ death. Echocardiography has also shown that a dilated left ventricular diameter (LVD) and decreased left ventricular ejection fraction (LVEF) occur in some patients with early septic shock, further suggesting that cardiac dysfunction may be present in patients with early septic shock. Until now, researchers and physician have generally recognized that cardiac dysfunction is closely related to patients’ prognosis. Therefore, improving cardiac function plays a crucial role in reducing mortality in patients with sepsis. However, the treatment for cardiac damage caused by sepsis is not ideal, because the mechanism of myocardial injury induced by sepsis is not clear.

CDKN1A is an important member of the cyclin-dependent kinase (CDK) inhibitor family and belongs to the Cip/Kip family of CDK inhibitors, hence the name p21^WAF1/CIP1^ (hereinafter referred to as p21)^3^. It is also an important downstream target of the tumor-suppressor p53^4^. p21 can regulate the cell cycle by inhibiting the binding of cyclin and CDK. p21 also plays an important role in cell senescence and apoptosis^5^. In mammals, p21 is rarely expressed in the embryonic and neonatal hearts but is highly expressed in the adult hearts.

A large number of studies have suggested that p21 is involved in the pathological process of myocardial injury, but the changes of p21 in cardiovascular disease and its effects are currently controversial. In different animal models of cardiac hypertrophy, the expression changes of p21 are inconsistent, and so are the corresponding effects. In Angiotensin II-mediated cardiac hypertrophy, p21 expression level is reduced, but in a variety of other cardiac hypertrophy models, such as spontaneously hypertensive rats and isoproterenol stimulation, p21 expression is continuously increased^6,7^. The expression of p21 in doxorubicin-induced cardiotoxicity is similar to that of cardiac hypertrophy and has its two sides^8,9^. Recently, it has been reported that p21 plays an important role in inflammatory diseases independently of cell cycle regulation, but research on p21 and sepsis-induced cardiac dysfunction has not been conducted.

In this study, we investigated the role of p21 in LPS-induced cardiac dysfunction and its molecular mechanism. We found that LPS-mediated autophagy increases improved inflammation and oxidative stress was partially dependent on p21. In addition, the effects of p21 on autophagy were achieved by the protein–protein interaction between p21 and microtubule-associated protein 1 light chain3 (LC3).

## Results

### p21 deficiency worsened cardiac dysfunction in LPS-treated mice

**Fig. 1:**
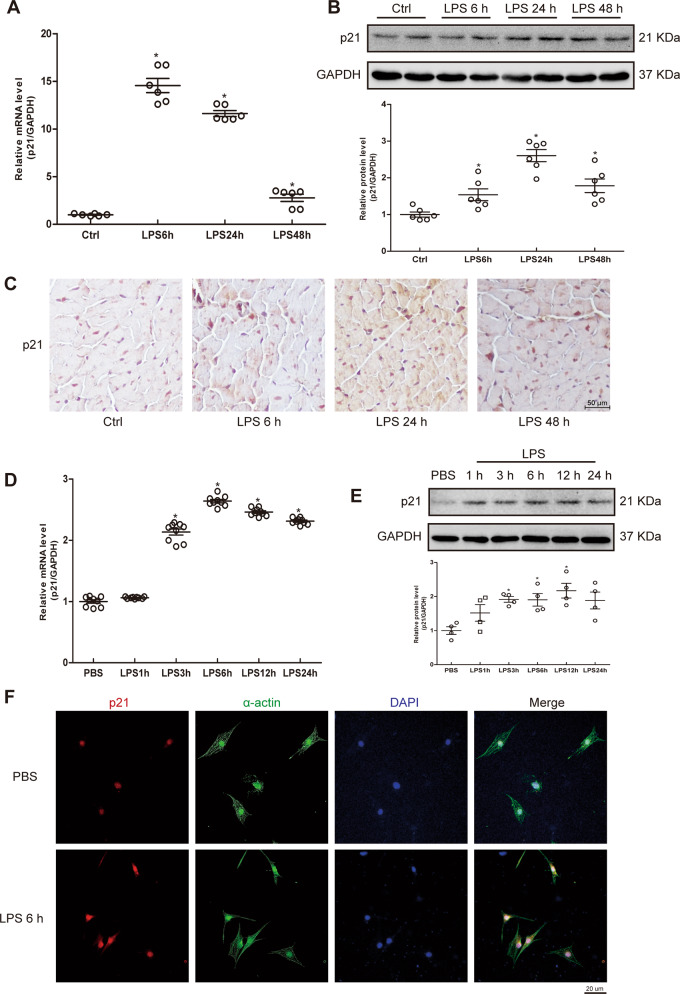
LPS induced the expression of p21. **a**–**c** The expression of p21 in the mouse heart. Relative mRNA level of p21 in the indicated WT mouse heart (**a**, *n* = 6). Representative western blot analysis of p21 (**b**, upper panel) and average fold change (**b**, lower panel) in the mouse hearts (*n* = 6). Representative immunohistochemical analysis of p21 in the indicated groups (**c**, *n* = 6). **d**–**f** The expression of p21 in NCRMs. Relative mRNA level of p21 in the indicated groups (**d**, *n* = 8). Representative western blot analysis of p21 (**e**, upper panel), and average fold change (**e**, *n* = 4, lower panel) in NCRMs before and after LPS stimulation. Representative immunofluorescence analysis of p21 (red fluorescence) and α-actin (green fluorescence) in the indicated groups (**f**, *n* = 6). Data are represented as mean ± SEM. *p < 0.05 versus Ctrl group, Ctrl indicates control.

To explore whether p21 was involved in the cardiac stress response after LPS stimulation, we first compared the mRNA and protein levels of p21. After LPS administration, mouse cardiac p21 mRNA and protein levels were significantly increased at 6, 24, and 48 h (Fig. 1a, b). Immunohistochemistry (IHC) showed that p21 was mainly expressed in the nucleus of myocardial tissues under basal condition, and p21 protein was increased in both the nucleus and cytosol after LPS stimulation (Fig. 1c). In addition, the administration of LPS to cardiomyocytes (CMs) increased mRNA and protein expression of p21 as well (Fig. 1d–f). These results suggest p21 may play a role in heart tissue after LPS stimulation.

**Fig. 2:**
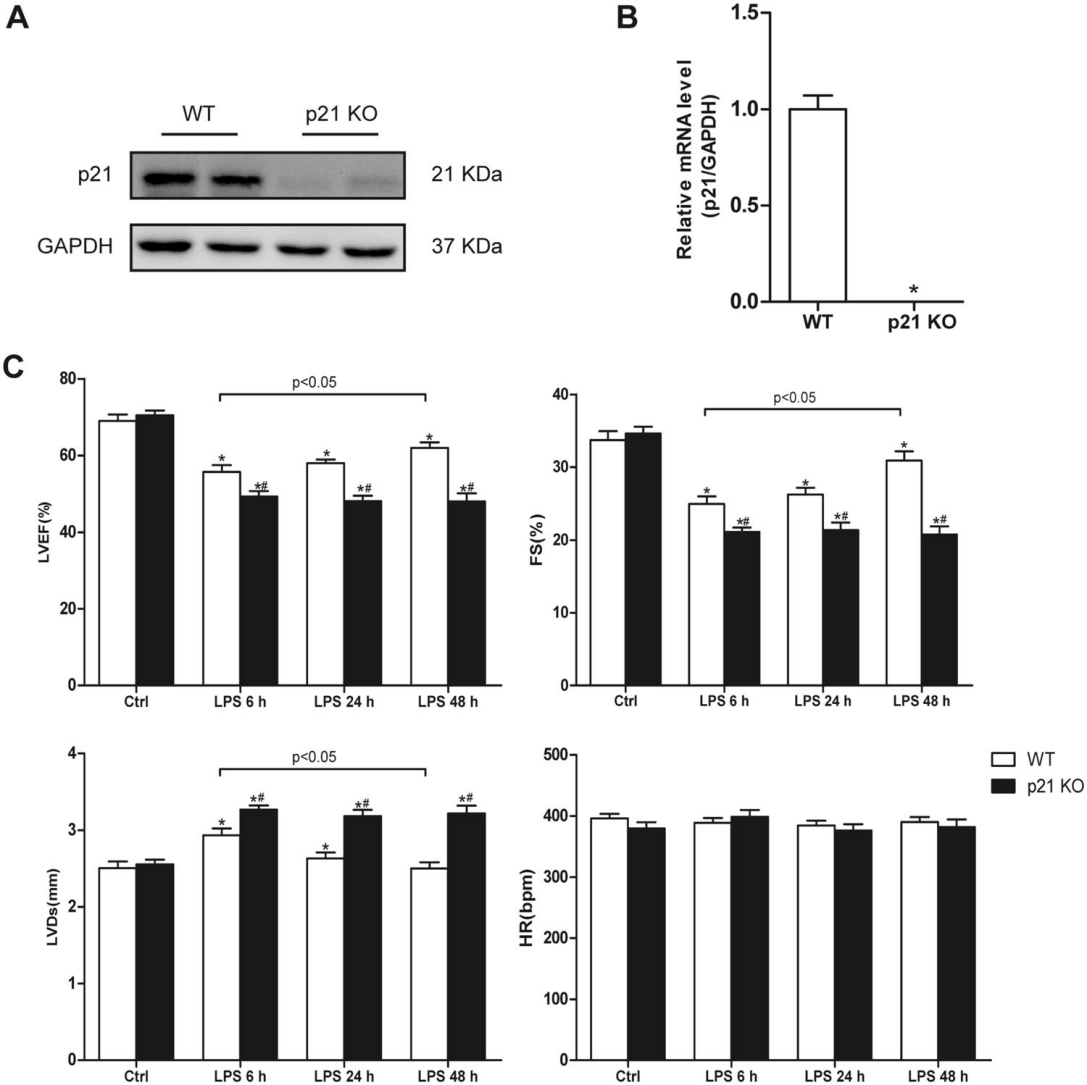
Identification of p21KO mice and characterization of cardiac function. **a**, **b** The expression of p21 between p21KO and WT mice. Representative western blot analysis of p21(**a**, *n* = 6) and relative mRNA level of p21 in the indicated mouse heart (**b**, *n* = 6). **c** Cardiac function was assessed by echocardiography in p21KO and WT mice (*n* = 8). Left ventricular ejection fraction (LVEF, upper panel) and fractional shortening (FS, upper panel); left ventricular end-systolic diameter ((LVDs, lower panel) and heart rate (HR, lower panel). *p < 0.05 versus Ctrl group, ^#^*p* < 0.05 versus corresponding LPS group.

We first identified cardiac mRNA and protein expression of p21 in wild-type (WT) and global knockout (p21KO) mice (Fig. 2a, b). Next, we investigated the role of p21 in cardiac dysfunction induced by LPS. The cardiac function was assessed by echocardiography in WT and p21KO mice after injection of saline or LPS (Fig. 2c). There was no significant difference in cardiac function between WT and p21KO mice under normal physiological conditions. After 6 h of LPS stimulation, both the WT and p21KO LPS groups showed significant cardiac dysfunction, manifesting as an obvious increase in LVDs and significant decreases in LVEF and fractional shortening. Compared with the WT LPS 6 h group, the changes of LVDs, LVEF, and EF in the p21KO LPS 6 h group were more pronounced. There were no significant differences in LVDs, LVEF, or EF between the LPS 6 h group and the LPS 24 h group in the WT mice, but the results showed a slight decrease in LVDs and a slight increase in LVEF and EF. The heart function of the p21KO LPS 24 h group was worse than that of the WT LPS 24 h group. Moreover, we found that the cardiac function of the WT LPS 48 h group did not reach a normal level, but it was significantly improved compared with the WT LPS 6 h group, while the cardiac function of the p21KO LPS 48 h group remained at a level similar to that of the p21KO LPS 6 h group. All of the data suggest that the loss of p21 exacerbates LPS-mediated cardiac dysfunction and delays recovery of cardiac function.

### p21 deficiency aggravated the inflammation, oxidative stress, and mitochondrial injury induced by LPS

**Fig. 3:**
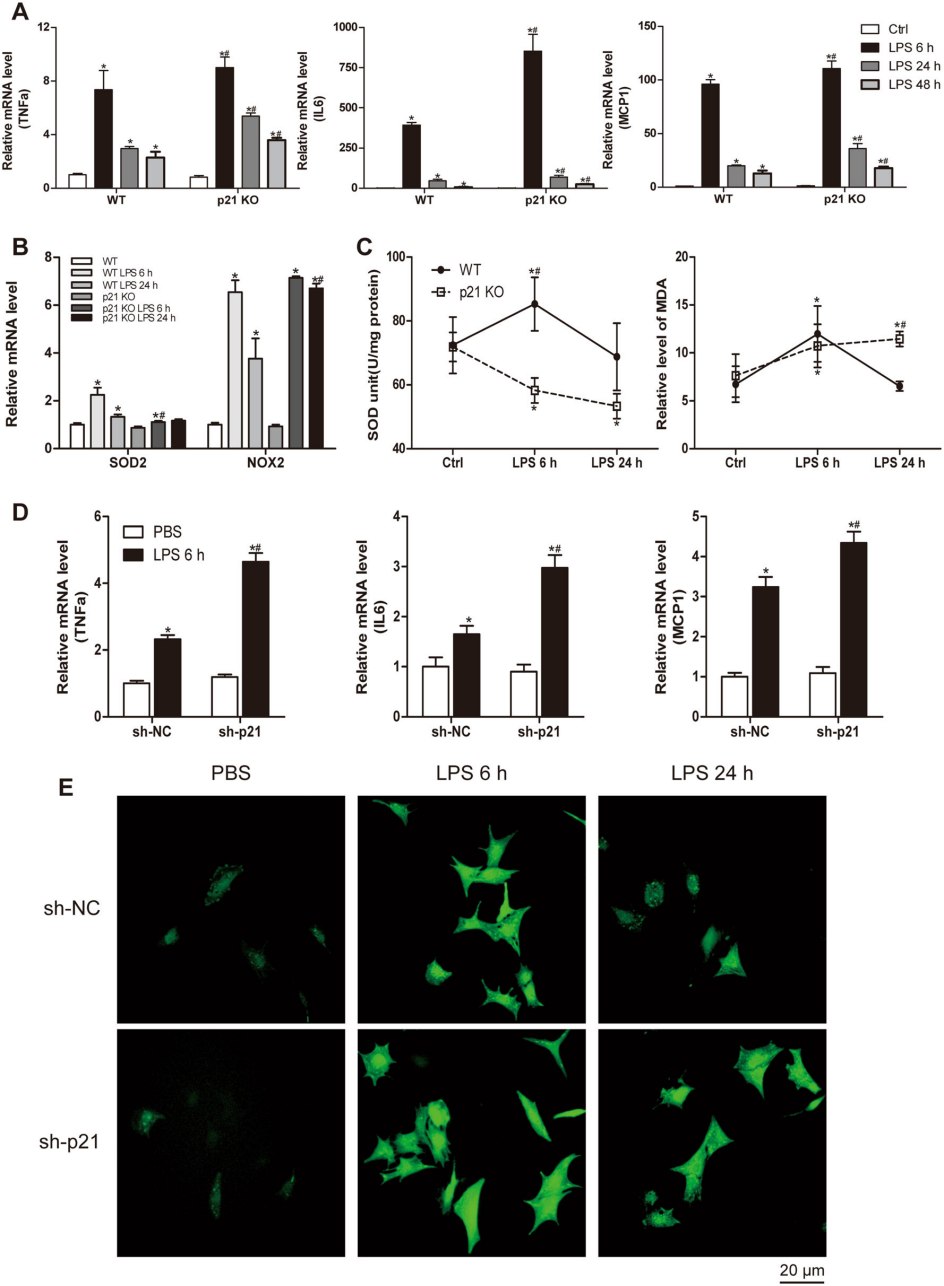
p21 deficiency exacerbated inflammatory and oxidative response caused by LPS. **a**, **b** Relative mRNA level of TNFα, IL6, and MCP1 (**a**) and SOD2 and NOX2 (**b**) in WT and p21KO mouse hearts (*n* = 6). **c** The activity of SOD (left panel, *n* = 6) and relative content of MDA (right panel, *n* = 6) in the indicated hearts of WT and p21KO mice. **d** Relative mRNA level of TNFα, IL6, and MCP1 in NCRMs (*n* = 6). **e** The level of ROS in the indicated groups of NCRMs (*n* = 6). *p < 0.05 versus Ctrl group, ^#^*p* < 0.05 versus corresponding LPS group.

The LPS-induced inflammatory response is one of the major causes of cardiac damage. To determine whether p21 deficiency worsened cardiac dysfunction by regulating inflammatory responses, we tested the mRNA levels of proinflammatory cytokines, including tumor necrosis factor-α (TNFα), interleukin-6 (IL6), and monocyte chemoattractant protein-1 (MCP1) in p21KO and WT mice under basal condition and in response to LPS (Fig. 3a). We found that the expression of these genes had no differences under basal conditions. LPS treatment increased the mRNA expression of TNFα, IL6, and MCP1 in both WT and p21KO mice, but the effect was markedly greater in p21KO mice. Next, we determined the effects of p21 downregulation on the LPS-induced increase of TNFα, IL6, and MCP1 expression in CMs (Fig. 3d). The results were similar to those obtained in cardiac tissue, with reduced myocardial expression of p21 significantly increasing TNFα, IL6, and MCP1 mRNA expression after LPS stimulation.

In addition to the inflammatory response, oxidative stress has also been shown to play an important role in LPS-induced cardiac dysfunction. Our results showed that mRNA levels of SOD2 and NOX2 did not differ between WT and p21KO mice at baseline (Fig. 3b). After LPS stimulation, the transcriptional levels of SOD2 were significantly elevated in WT mice (Fig. 3b). The SOD2 mRNA level was slightly increased in the p21KO LPS group compared with the WT group, but it was lower than that of the WT LPS group (Fig. 3b). In addition, the activity of SOD in the myocardial tissue of p21KO mice remained at baseline at 6 and 24 h post-LPS stimulation, while that in WT mice was markedly increased at 6 h after LPS stimulation, then it returned to the basal level at 24 h (Fig. 3c). Compared with the WT group, the cardiac NOX2 mRNA level was increased after LPS stimulation, and it was further upregulated by p21 deficiency (Fig. 3b).

Malondialdehyde (MDA) assays showed that the MDA content in the myocardial tissue of p21KO mice was significantly increased at 6 and 24 h after LPS stimulation compared to the WT group, while the MDA content in the myocardial tissue of WT mice stimulated by LPS was significantly increased at 6 h, and then it decreased to a normal level at 24 h (Fig. 3c). We also examined the effect of decreased p21 expression on the LPS-induced reactive oxygen species (ROS) level in CMs. The data further confirmed p21 deficiency exacerbated myocardial injury induced by LPS (Fig. 3e).

**Fig. 4:**
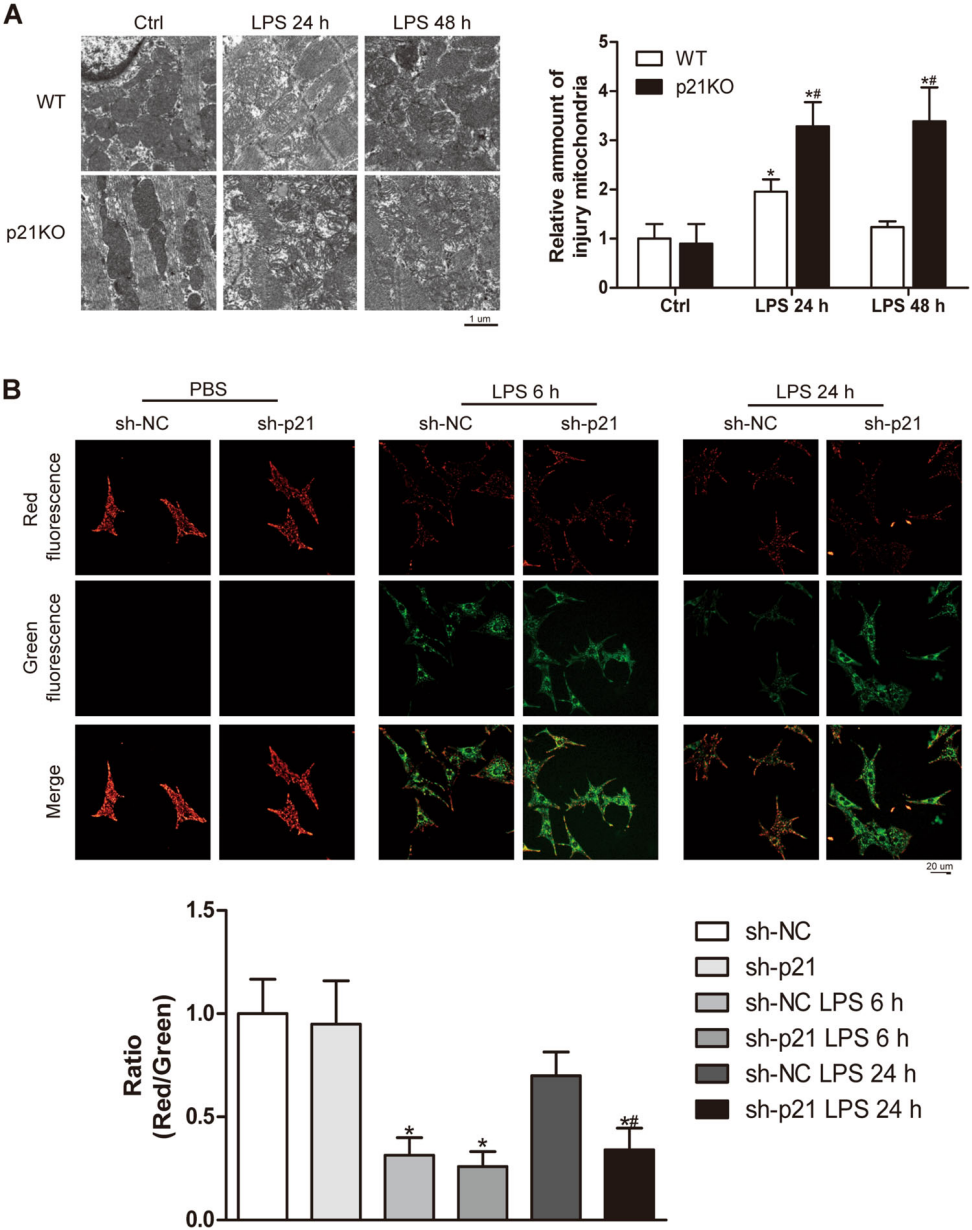
p21 deficiency aggravated LPS-induced mitochondrial injury. **a** Representative transmission electron microscopic images of mitochondria (left panel) and quantification of the injured mitochondria (right panel) in the indicated heart tissue groups (*n* = 6). **b** Representative images of JC1 staining (upper panel) and quantitative analysis of the shift of mitochondrial red fluorescence to green fluorescence (lower panel) in NRCMs (*n* = 6). *p < 0.05 versus Ctrl group, ^#^*p* < 0.05 versus corresponding LPS group.

Mitochondria are the main organelles involved in oxidative stress, and it has been reported that LPS induces mitochondria injury. Therefore, we observed mitochondrial changes between the WT and p21KO groups. Electron microscopy showed that, after LPS stimulation for 24 h and 48 h, in both the WT and p21KO mice we observed cardiac mitochondrial swelling and the ridge structure disappearing (Fig. 4a). Compared with that of the corresponding stimulated time in WT mice, p21 deficiency resulted in an increased number of abnormal structure of mitochondria, and similar results were obtained in vitro (Fig. 4a, b). In addition to being involved in oxidative reactions, mitochondria are also important organs for maintaining the energy supply to the heart. We therefore compared the transcription levels of energy-related factors between WT and p21KO mice, but the results suggested that p21 deficiency did not affect the expression of these genes (Fig. S1).

Collectively, these results indicate that a lack of p21 aggravates LPS-induced cardiac inflammation, oxidative stress, and mitochondria damage.

### p21 regulated autophagy level

**Fig. 5:**
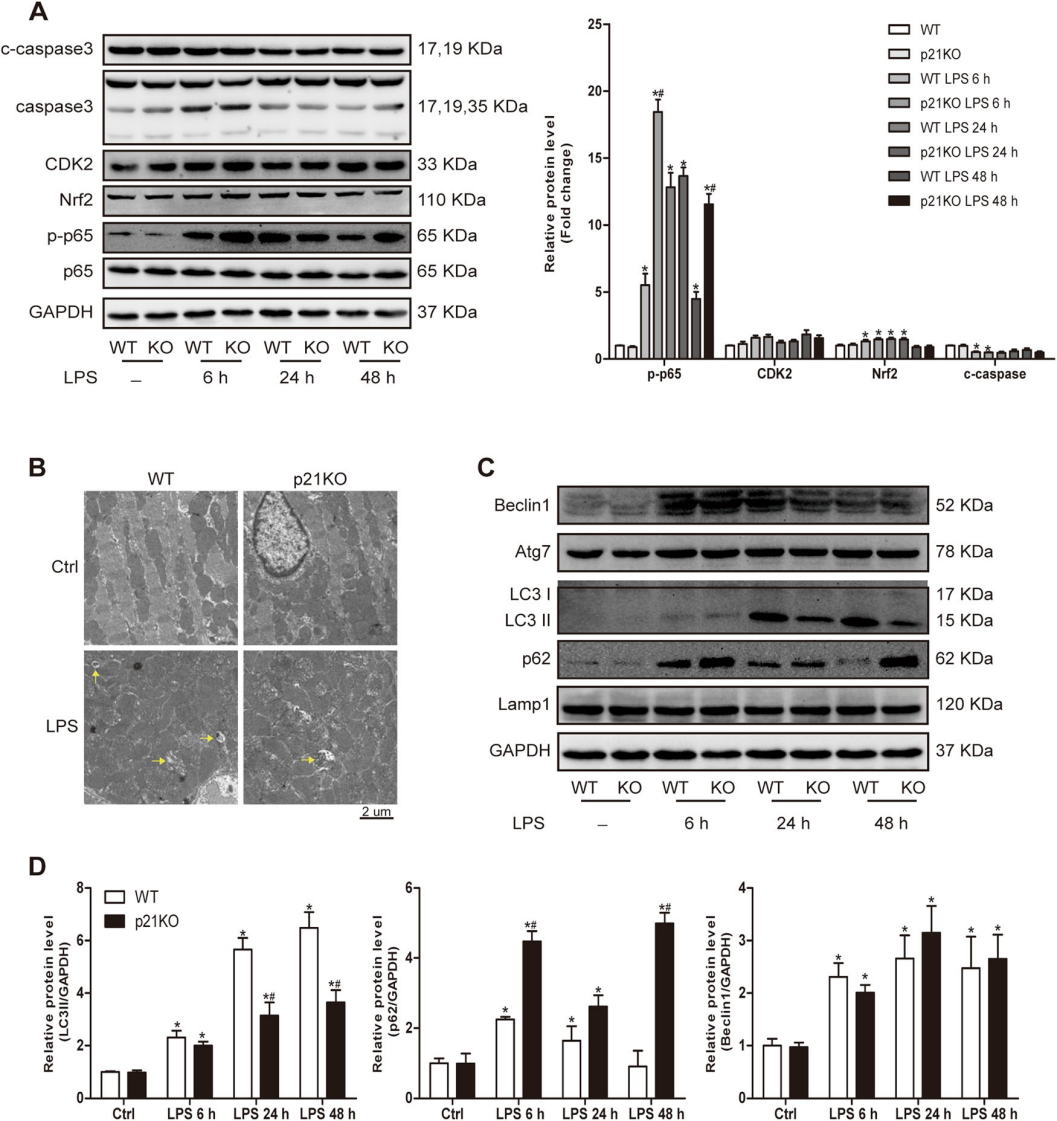
p21 deficiency impaired LPS-induced autophagy increase. **a** Representative western blot analysis of proteins involved in inflammation, oxidative stress, and apoptosis (left panel) and average fold change (right panel) in the indicated hearts (*n* = 6). **b** Representative transmission electron microscope images of heart tissue. Yellow arrows indicate autophagosomes (*n* = 6). **c** Representative western blot analysis of proteins involved in autophagy flux in the indicated hearts of WT and p21KO mice. **d** Quantization of protein level of LC3II, p62, and Beclin1 in mouse hearts (*n* = 6). *p < 0.05 versus Ctrl group, ^#^*p* < 0.05 versus corresponding LPS group.

To explore the molecular mechanism of how p21 regulates inflammation and oxidative stress, we compared the proteins involved in the above pathways between WT and p21KO mice. The results showed that antioxidative protein Nrf2, which was increased after LPS administration, did not differ between WT and p21KO mice (Fig. 5a). In addition, p21 did not affect the protein expression of cycle regulatory protein CDK2 or apoptotic executive protein cleaved-caspase3 after injection of saline or LPS (Fig. 5a). However, we found that the level of p-p65 was significantly increased in p21KO mice compared to WT mice after LPS stimulation (Fig. 5a).

The above results showed that p21 deficiency could increase the number of damaged mitochondria. The increase in damaged mitochondria may be due to either too much production or not enough clearance. It has been proven that autophagy is one of the main ways to clear damaged mitochondria. Therefore, we observed the autophagy level between WT and p21KO mice at 24 h post-LPS stimulation. The results from electron microscopy showed that LPS caused an increase of autophagosomes in WT mice, while the increase was impaired in p21KO mice (Fig. 5b).

**Fig. 6:**
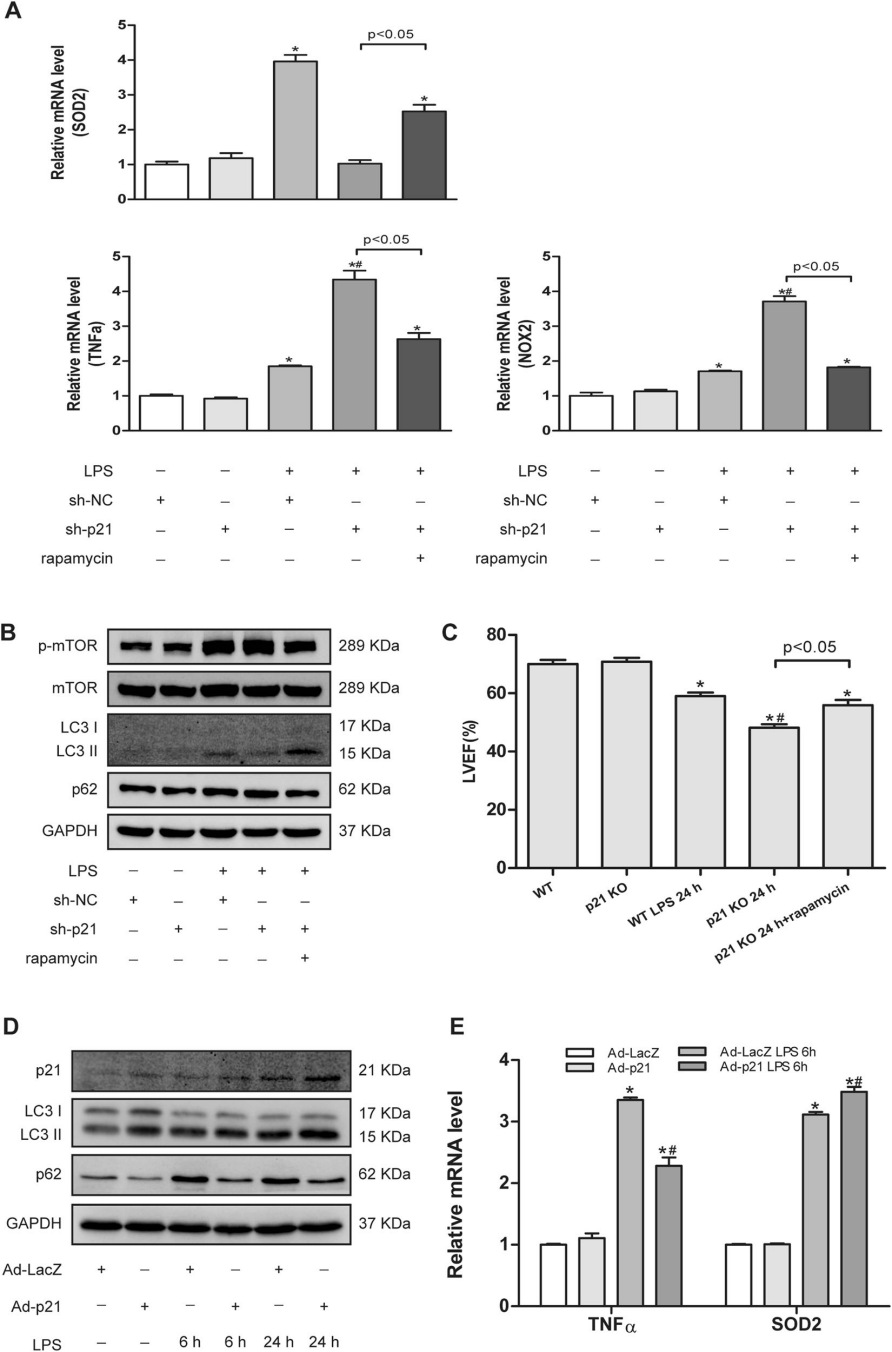
Autophagy activation improved cardiac damage. **a**–**c** Autophagy inducer rapamycin attenuated cardiac injury induced by p21 downregulation. Relative mRNA level of TNFα, SOD2, and NOX2 in the indicated groups of NRCMs (**a**, *n* = 6). Representative western blot analysis of p-mTOR, mTOR, LC3B, p62, and GAPDH in NRCMs (**b**, *n* = 6). Cardiac EF was measured by echocardiography in the indicated mouse hearts (**c**, *n* = 6). **d**, **e** p21 overexpression activated autophagy flux and attenuated inflammation and oxidative stress induced by LPS. Representative western blot analysis of p21, LC3, p62, and GAPDH in NRCMs (**d**, *n* = 6) and relative mRNA levels of TNFα and SOD2 in NRCMs (**e**, *n* = 6). *p < 0.05 versus Ctrl group, ^#^*p* < 0.05 versus corresponding LPS group.

**Fig. 7:**
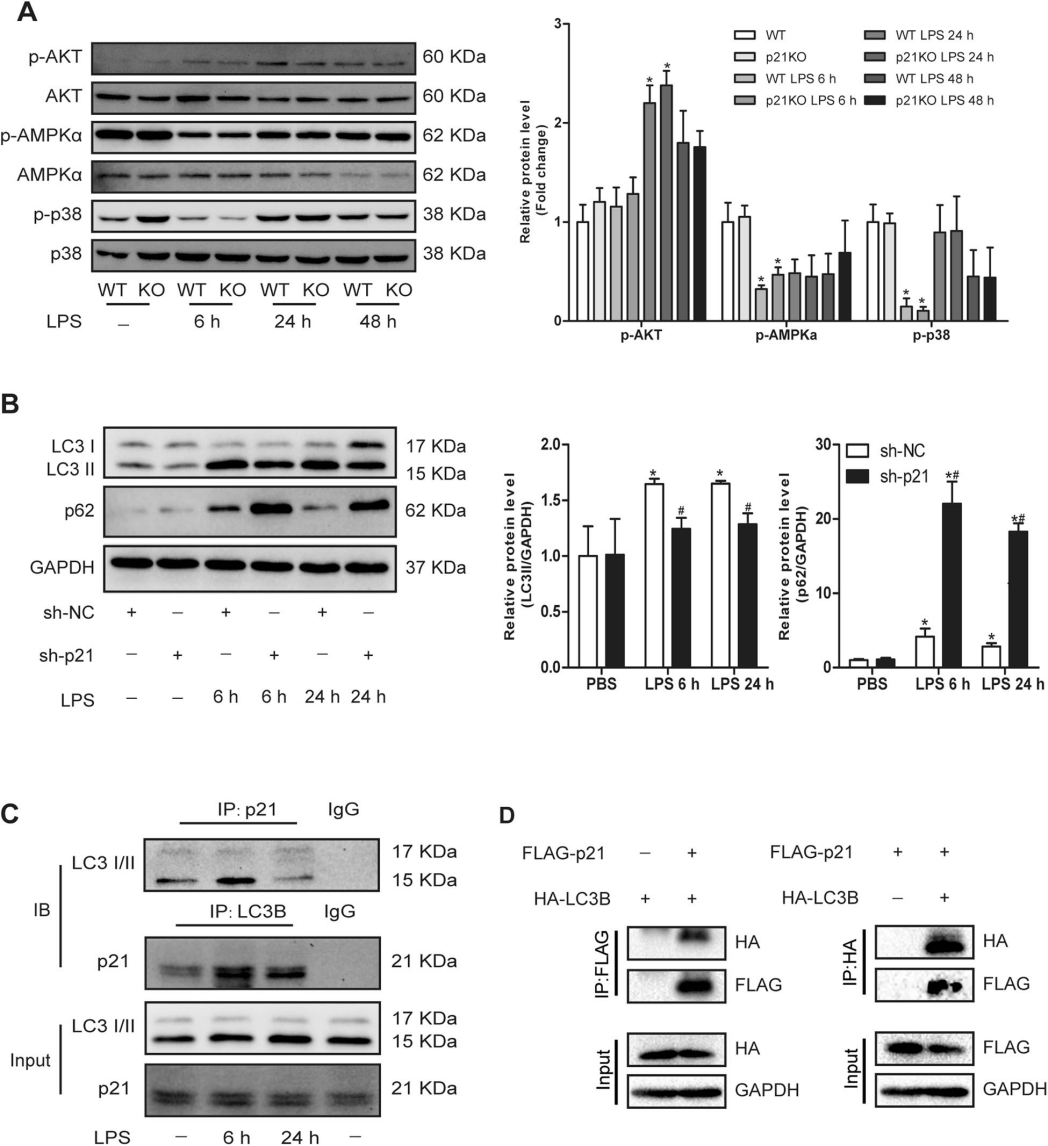
p21 regulates LC3II by interacting with LC3. **a** Representative western blot analysis of the upstream kinases associated with LPS-induced autophagy (left panel) and average fold change (right panel) in the indicated hearts (*n* = 6). **b** Representative western blot analysis of LC3, p62, and GAPDH (left panel) and average fold change (right panel) in NRCMs (*n* = 6). **c** CoIP assays to test the interaction between endogenous p21 and LC3 in the indicated groups of NRCMs (*n* = 3). **d** CoIP assays to test the interaction between exogenously expressed p21 and LC3 in HEK293T cells. FLAG-tagged p21 or HA-tagged LC3B were overexpressed in HEK293T cells (*n* = 3). *p < 0.05 versus Ctrl group. ^#^*p* < 0.05 versus corresponding LPS group.

Moreover, we also tested the protein levels of LC3 and p62. In line with the previous results, the cardiac level of LC3II was reduced and p62 was increased in p21KO mice compared to WT mice after LPS administration (Fig. 5c, d), and the same results were obtained in neonatal rat CMs (NRCMs; Fig. 7b). To verify that p21 deficiency exacerbates LPS-induced cardiac injury due to impaired autophagy activation, rapamycin, an autophagy agonist, was added. We found that rapamycin could significantly improve the cardiac dysfunction caused by p21 deficiency in LPS-treated mice (Fig. 6c). In addition, we found that the administration of rapamycin could improve an impaired autophagy flux and attenuate the increase of TNFα and NOX2 and the decrease of SOD2 caused by p21 deficiency in CMs, further indicating that increasing autophagy levels improves cardiac outcomes with p21 deficiency (Fig. 6a, b). We also found that the loss of p21 did not affect the expression of phospho-mammalian target of rapamycin (p-mTOR) in CMs (Fig. 6b).

Next, we examined whether the overexpression of p21 caused an increase in autophagy in CMs. The results showed that, compared with the control group, p21 overexpression could increase the level of LC3II in the basal state, and after LPS stimulation, it could activate an autophagy flux (Fig. 6d). In addition, we also found that p21 overexpression reduced the increase of TNFα, and it promoted the increase of SOD2 caused by LPS (Fig. 6e).

All of these results indicate that p21 could regulate the level of LC3II and then influence the increased autophagy level caused by LPS.

### p21 modulated LC3II by interacting with LC3B

The expression level of LC3II is affected by many factors; thus we studied how p21 regulates LC3II. We first examined changes in the expression levels of the upstream kinases associated with LPS-induced autophagy. However, the data indicated that there were no differences in the protein levels of p-AKT, p-AMPK, or p-p38 between WT and p21KO mice (Fig. 7a). Then we tested Beclin1 and Atg7, which directly regulate the formation of autophagy, and the results showed that p21 deficiency did not affect these proteins in the absence or presence of LPS (Fig. 5c). We also found that p21 deficiency did not affect the expression of Lamp1 (Fig. 5c), indicating that the decrease of LC3II may not be due to the increase in autophagic lysosomes. Since p21 did not affect the upstream or downstream regulators of autophagy, we hypothesized that perhaps p21 directly regulates LC3B. Then we examined the physical interaction between p21 and LC3B in CMs. Coimmunoprecipitation (CoIP) assays revealed that endogenous p21 and endogenous LC3B interact with each other in NRCMs (Fig. 7c). In addition, reciprocal CoIP experiments in HEK293T cells confirmed the interaction between p21 and LC3B (Fig. 7d).

## Discussion

**Fig. 8:**
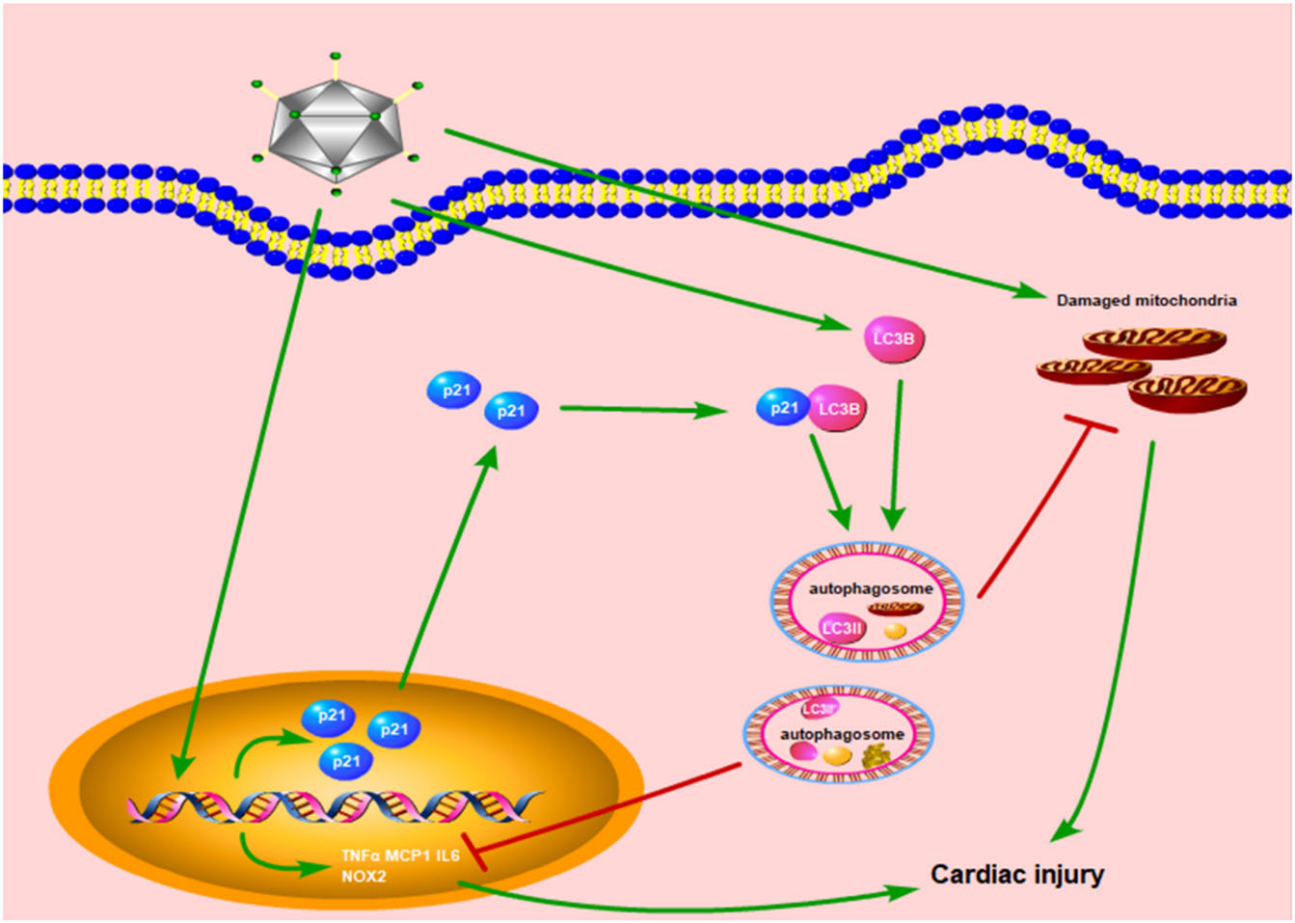
A diagram of the proposed pathway for the cardioprotective of p21. LPS induces the expression of p21. p21 binds to LC3B and regulates cardiac autophagy level to attenuate the increase expression of TNFα, MCP1, IL6 as well as damaged mitochondria induced by LPS stimulation, thus protecting the heart.

In this study, we identify that p21 could regulate the cardiac autophagy level, thus protecting the heart against LPS-induced inflammatory and oxidative injury (Fig. 8). In addition, we found that p21 can bind to LC3B and regulate the formation of LC3II.

It has been reported that the expression level of p21 in macrophages is significantly increased after LPS stimulation and that p21 is involved in the anti-inflammatory effect of macrophages^10,11^. It is well known that macrophages are proliferating cells, and the primary biological function of p21 is to regulate the cell cycle. Interestingly, p21 is also highly expressed in CMs, which are nonproliferating cells. This raises the question of whether p21 in CM, one of the target cells of inflammatory responses, change in response to inflammatory stimulation. Our experimental results showed that the expression level of p21 presented dynamic changes during LPS stimulation, but it was significantly higher than the basal state, which suggests that p21 might play a role in LPS-mediated cardiac injury. Moreover, the results of cardiac function tests further demonstrated that p21 was involved in LPS-induced cardiac dysfunction.

The toxic effect of LPS is mainly induced by the inflammatory response. Previous studies and our studies have confirmed that LPS promotes the inflammatory response, and the absence of p21 aggravates the production of inflammatory factors. In the LPS-mediated inflammatory model, studies have shown that p21KO mice are more prone to endotoxin shock after LPS stimulation, because p21 in macrophages inhibits the formation of IL1β through its CDK-binding domain, thereby inhibiting inflammation and alleviating the deterioration of endotoxemia^12^. In addition, p21 can also regulate macrophage polarization in an LPS second-stroke mice model^11^. These studies showed that p21 has anti-inflammatory effects, but they focused on its role in inflammatory cells, which are proliferating cells. We found that p21 knockdown promoted the expression of proinflammatory factors in vivo and in vitro, whereas p21 overexpression relieved their increased mRNA levels in primary CM treated with LPS, suggesting that p21 regulates the anti-inflammatory effect of nonproliferating cells as well. In addition, the activation of p65 may partly account for the inflammatory-regulation effects of p21, which is in accordance with previous research.

Increased ROS is another important manifestation of heart dysfunction induced by LPS. LPS can promote the formation of ROS, which further aggravates myocardial injury^13,14^. By examining the transcription level of antioxidant enzymes SOD2 and NOX2 in myocardial tissue and in CM stimulated by LPS, we found that p21 deletion could promote the expression of NOX2 and inhibit the transcription of SOD2. In addition, our detection of SOD activity and MDA content in cardiac tissue, as well as direct observations of ROS in CM, further supports the fact that p21 deficiency contributes to ROS production induced by LPS. However, p21 overexpression promoted increased SOD2 influenced by LPS, demonstrating that p21 has the ability to alleviate oxidative stress.

Previous studies have shown that p21 could protect cells from the toxic effects of various compounds by inhibiting the production of ROS^15,16^. It has been reported that, when fibroblasts and HCT-116 cells suffer oxidative stress injury, p21 directly binds to Nrf2 and promotes the antioxidant effect of Nrf2, which suggests that p21 could regulate oxidative stress injury^17^. However, in this study we found that p21 did not influence Nrf2, implicating that p21 regulate oxidative stress independently of Nrf2 pathway.

In addition to the direct damage of inflammatory cytokines and ROS to the heart, an insufficient energy supply in the heart accounts for an important aspect of LPS-induced cardiac dysfunction^18,19^. LPS inhibits the expression of CD36^20^ and the expression of lipid oxidation genes^21^, thereby reducing the formation of ATP and leading to cardiac dysfunction. In this study, we detected the transcriptional level of energy-related genes in heart tissue and found that p21 deletion had no effect on LPS-induced myocardial energy inhibition, indicating that deterioration of cardiac function caused by p21 deletion may not be related to energy metabolism.

Increasing evidences has suggested that sepsis induces increased autophagy in multiple organs, including the heart. Increased autophagy has been reported to play a protective role in septic cardiac dysfunction^22,23^. Notably, p21 also plays an important role in the regulation of autophagy. The DNA-damaging agent 5-methoxyflavone can induce apoptosis and autophagy in HCT-116 human colon cancer cells via a p21-dependent pathway^24^. In addition, the antimalarial drug quinacrine induces autophagy and apoptosis of breast cancer cells by regulating p21, thereby achieving the effect of antitumor cell proliferation^25^. All of these results suggest that p21 induces the formation of autophagy and exerts an antitumor effect. Our study found that, after LPS stimulation, autophagy levels gradually increased with the prolonged stimulation time in cardiac tissues and CM, but after reaching the peak, autophagy levels showed a decreasing trend, and the corresponding cardiac function also deteriorated significantly in the early stage but then gradually improved. After p21 deletion, compared with the normal group, the level of autophagy in the myocardial tissue and primary CM was decreased, and mouse cardiac function was always in a dysfunctional state. These results suggest that the absence of p21 may lead to insufficient autophagy in CM, which then cannot timely remove the harmful stimuli and damaged organelles from within the cells, thus preventing their recovery of cardiac function. The increasing autophagy level decreased the cardiac injury caused by p21 deletion, which further demonstrated that the effects of p21 on cardiac damage induced by LPS are partially mediated through regulating the autophagy level.

To clarify the specific mechanism of the impaired autophagy increase caused by p21 deficiency, we first examined the expression of related kinases acting upstream of autophagy. mTOR is currently recognized as an important molecule in upstream inhibition of autophagy formation^26^. Studies have suggested that mTOR activation may suppress beneficial autophagy activity during severe stages of sepsis, but activation of autophagy in early sepsis may be independent of mTOR^23^, which is in line with our results in this study. Moreover, mTOR is a direct target of AMPKα, and AMPKα can promote the formation of autophagy by inhibiting the activity of the mTOR complex^27^. Our previous study has proven that p21 activates autophagy by regulating AKT and AMPKα in the hypertrophic heart^28^. In this study, we found that, after LPS stimulation, the levels of AMPKα were significantly decreased while the level of autophagy increased, indicating that the increase in LPS-induced autophagy was not mediated through the activation of AMPKα. In addition, our results also pointed out that p21 deletion did not affect the level of p-AMPKα and p-AKT after LPS stimulation. Recently, a novel mTOR-independent autophagy pathway has been reported. p38 plays an important role in autophagy regulation through p38 mitogen-activated protein kinase interacting protein (p38IP)^29^. In addition, it has been reported that Toll-like receptor 4 activation can improve liver injury by inducing increased autophagy through the p38 signaling pathway^30^. These results indicate that the p38 signaling pathway directly regulates intracellular autophagy levels. Our study confirmed that the expression level of p-p38 was significantly increased after LPS stimulation, but the expression level of p-p38 was not significantly changed after p21 deletion, suggesting that p21 does not regulate the level of autophagy through p38 pathway.

p21 does not affect autophagy formation by affecting autophagy upstream kinases, thus we wondered whether p21 directly affects autophagy-related proteins. It has been reported that C2-ceramide could reduce the expression of the autophagy-associated proteins Beclin1 and Atg5 by p21, thereby inhibiting the level of autophagy in mouse embryonic fibroblasts^31^. In this study, we also detected expression changes of Beclin1 and Atg7 in WT and p21KO mice after LPS stimulation, but there was no significant difference between the two groups, suggesting that p21 also does not act by regulating the initiation of autophagy. The excessive activation of autophagy lysosomes may also cause a decrease in autophagy level. However, the results of our study showed that p21 deletion resulted in aggregation of autophagy substrate p62 and had no significant effect on the expression of lysosomal-related protein Lamp1, indicating that p21 also does not affect the degradation of the autophagosome.

In the process of autophagy, the formation and maturation of autophagosomes are regulated by several core proteins related to autophagy in a highly controllable manner. The LC3II level has become one of the gold standards for the detection of intracellular autophagy^32^. Like other Atg proteins, LC3 is tightly regulated at the transcriptional, posttranscriptional, or posttranslational levels. According to some reports, transcription factors such as ATF4, CHOP, E2F1, FOXO 1/3, GATA1, and ZKSCAN3 have been found to regulate the LC3 expression level^33^. This is the first report to show that p21 directly binds to LC3, and the absence of p21 led to significantly impaired increase of LC3II in response to LPS stimulation, suggesting that p21 may directly regulate the formation of LC3II by binding to LC3. Notably, we did not explore how LC3 expression was regulated after p21 bound to LC3, which will be investigated in the future.

## Methods

### Animals and treatment

p21^CIP1/WAF1^ global knockout mice (016565, B6.129S6-Cdkn1atm1Led/J, p21KO) were purchased from The Jackson Laboratory (Bar Harbor, USA) and C57BL/6J WT mice were purchased from Institute of Laboratory Animal Science, Chinese Academy of Medical Sciences (Beijing, China). Male mice aged 8–12 weeks weighing 23–25 g were used. All procedures were conducted in accordance with the Guidelines for the Care and Use of Laboratory Animals published by the United States National Institutes of Health (NIH Publication, revised 2011) and were approved by the Chinese Animal Welfare Committee as well as Wuhan Renmin Hospital.

To establish a mouse nonlethal septic cardiac dysfunction model^34^, WT and p21KO mice were intraperitoneally injected with a single dose of 5 mg/kg *Escherichia coli* LPS (serotype 0111: B4, Sigma) dissolved in sterile saline, and control mice received an equal volume of saline. p21 KO mice or WT mice were divided into four groups, and they are control group, LPS 6 h group, LPS 24 h group, and LPS 48 h group, with at least 20 mice in each group. Echocardiography was performed at 6, 24, or 48 h after LPS challenge to assess mouse cardiac function. To induce autophagy, the autophagy agonist rapamycin (6 mg/kg, Sigma) was intraperitoneally injected 30 min before LPS treatment, as previously reported^27^. Mice were sacrificed by cervical dislocation, and their hearts were rapidly excised, weighed, and collected for later use. All animal experiments were replicated at least three times.

### Adenoviral vector construction

An adenoviral vector carrying Rat p21 small hairpin RNAs (sh-p21) to knockdown p21 was generated by the OBIO Technology Corp., Ltd. (Shanghai, China), and scrambled shRNA (sh-NC) was used as the control. Adenovirus vector carrying Rat p21 (Ad-p21) to overexpress p21 and empty carrier control Ad-LacZ were constructed and amplified by the iBioscience Company (Jinan, China).

### Plasmid construction

For constructing the pcDNA5-FLAG-p21 and pcDNA5-HA-LC3B recombinant plasmids, the full-length human p21 and LC3B-coding regions were amplified from the cDNA of HEK293T cells by polymerase chain reaction (PCR). The pcDNA5-FLAG and pcDNA5-HA plasmids were digested with BamH I (FD0054, Thermo) and Xho I (FD0694, Thermo). The PCR products of p21 and LC3B were ligated to the digested pcDNA5-FLAG and pcDNA5-HA plasmids, respectively, using the recombinase (C112–01, Vazyme) according to the manufacturer’s instruction. The primers for amplifying the target gene are listed below: human-p21-F: TCGGGTTTAAACGGATCCATGTCAGAACCGGCTGGGGA, human-p21-R: GGGCCCTCTAGACTCGAGTTAGGGCTTCCTCTTGGAGAAGATCAG, human-LC3B-F: TCGGGTTTAAACGGATCCATGCCGTCGGAGAAGACCTT, human-LC3B-R: GGGCCCTCTAGACTCGAGTTACACTGACAATTTCATCCCGAACGT.

### Cell culture and treatment

Primary CMs (NRCMs) were isolated from the left ventricle of neonatal SD rats and cultured as described previously^35^. NRCMs were incubated in serum-free medium for 12 h before LPS stimulation. Then the medium was replaced with Dulbecco’s modified Eagle’s medium (DMEM)/F-12 (11330, GIBCO) containing 15% fetal bovine serum (FBS; 10099, GIBCO), penicillin (100 U/mL), and streptomycin (100 mg/mL) (15140, GIBCO), and NRCMs were challenged with LPS (10 µg/mL). The autophagy agonist rapamycin (200 nM, Sigma) was used 2 h prior to LPS exposure to induce autophagy^36^. All controls received appropriate vehicles. To knockdown or overexpress p21, NRCMs were transduced with the corresponding adenoviruses at a multiplicity of infection of 100 for 24 h before LPS administration.

HEK293T cells were purchased from the Cell Bank of the Chinese Academy of Sciences (Shanghai, China) and cultured in DMEM (11885, GIBCO) supplemented with 10% FBS. HEK293T cells were cotransfected with plasmids encoding HA-LC3B and/or FLAG-p21 according to the manufacturer’s instructions. After transfection for 24 h, HEK293T cells were harvested for IP. All cell experiments were repeated at least three times.

### Immunoblot analysis

For western blots, hearts and NCRMs were lysed in RIPA lysis buffer (G2002, Wuhan Google Biological Technology Co., Ltd, China) containing phenylmethanesulfonylfluoride (1 mM, Wuhan Google Biological Technology Co., Ltd, China) and protease inhibitor pellets (Roche), and the protein was isolated as previously described^20^. Equal amounts of proteins were separated by 10–12% sodium dodecyl sulfate-polyacrylamide gel electrophoresis. The antibodies used were: anti-LC3B (1:1000, CST, 3868), anti-p62 (1:1000, CST, 23214), anti-p21 (1:200, Santa Cruz Biotechnology, sc-6246), anti-p-AKT (1:1000, CST, 4060), anti-AKT (1:1000, CST, 4691), anti-p-mTOR (1:1000, CST, 2971), anti-mTOR (1:1000, CST, 2983), anti-p-AMPKα (1:1000, CST, 2535), anti-AMPKα (1:1000, CST, 2603), anti-p-p38 (1:1000, CST, 4511), anti-p38 (1:1000, CST, 9212), anti-p-p65 (1:1000, CST, 3033), anti-p65 (1:1000, CST, 8242), anti-Beclin1 (1:1000, Abcam, ab62557), anti-Atg7 (1:1000, CST, 2613), anti-Lamp1 (1:1000, Abcam, ab13523), anti-cleaved-caspase3 (1:1000, CST, 9661), anti-caspase3 (1:1000, CST, 9662), anti-CDK2 (1:1000, CST, 2546), anti-Nrf2 (1:1000, Abcam) and anti-GAPDH (1:1000, CST, 2118).

For IP, NRCMs or HEK293T cells were lysed with ice-cold IP buffer containing Tris HCl (20 mM, pH 8.0), NaCl (137 mM), NP-40 (0.5%), EDTA (0.5 mM), and a protease inhibitor cocktail. Then the cell lysate was incubated with the indicated antibodies overnight at 4 °C. Subsequently, Protein A/G-agarose beads were added and incubated for 2 h. After IP, the immunocomplexes were washed with cell lysis buffer five times, then boiled at 100 °C for 5 min and subjected to immunoblotting.

### Real-time PCR analysis

Total RNA was extracted from heart tissues or NRCMs using Trizol reagent (15596–026, Invitrogen), and then equal amounts of total RNA (2 μg) were reverse-transcribed into cDNA using a Transcriptor First-Strand cDNA Synthesis Kit (04896866001, Roche), according to the manufacturer’s protocol. LightCycler 480 SYBR Green I Master Mix (04707516001, Roche) was used to amplify and quantify the PCR products. Relative mRNA expression of the target genes was normalized to glyceraldehyde 3-phosphate dehydrogenase (GAPDH). Specific primer sequences used in the experiments are listed in Table 1.

**Table 1:** Primers used for real-time RT-PCR.

Gene	Species	Forward primer (5′ → 3′)	Reverse primer (5′ → 3′)
TNFα	Mouse	ACTGAACTTCGGGGTGATCGGT	TGGTTTGCTACGACGTGGGCTA
IL6	Mouse	AGTTGCCTTCTTGGGACTGA	TCCACGATTTCCCAGAGAAC
MCP1	Mouse	TGGCTCAGCCAGATGCAGT	CCAGCCTACTCATTGGGATCA
NOX2	Mouse	TTCCAGTGCGTGTTGCTCGACA	TGGCGGTGTGCAGTGCTATCAT
SOD2	Mouse	CCGTCCGTGTCGCCGTCCTC	GCCGCGTGGTGCTTGCTGTG
GAPDH	Mouse	ACTCCACTCACGGCAAATTC	TCTCCATGGTGGTGAAGACA
TNFα	Rat	AGCATGATCCGAGATGTGGAA	TAGACAGAAGAGCGTGGTGGC
IL6	Rat	GTTGCCTTCTTGGGACTGATG	ATACTGGTCTGTTGTGGGTGGT
MCP1	Rat	AGTCGGCTGGAGAACTACAAGA	CTGAAGTCCTTAGGGTTGATGC
SOD2	Rat	AGCCTCCCTGACCTGCCTTA	CGCCTCGTGGTACTTCTCCTC
NOX2	Rat	TGAATCTCAGGCCAATCACTTT	AATGGTCTTGAACTCGTTATCCC
GAPDH	Rat	GACATGCCGCCTGGAGAAAC	AGCCCAGGATGCCCTTTAGT

### Histological analysis

For IHC, paraffin-embedded heart tissue sections were incubated with primary antibody against p21 (1:50, Santa Cruz Biotechnology, sc-6246) overnight at 4 °C. Horseradish peroxidase-conjugated secondary antibody (1:200) was applied to the heart tissue and diaminobenzidine was used as the substrate.

For immunofluorescence staining, NRCMs were cultured on cover slips. After different treatments, NRCMs were fixed with 4% paraformaldehyde and permeabilized with 0.03% Triton®X-100. Then NRCMs were incubated with specific antibody overnight at 4 °C. After washing three times, the NRCMs were incubated with a secondary antibody conjugated to Alexa-488 or -568 (1:200). Finally, 4′,6-diamidino-2-phenylindole was used to stain the nuclei. The NRCMs were observed under an Olympus fluorescence microscope.

### Measurement of oxidative stress

The activity of cardiac SOD was measured by a Total Superoxide Dismutase Assay Kit with WST-8 (Beyotime Biotechnology, China), and the content of cardiac MDA was determined by a Lipid Peroxidation MDA Assay Kit (Beyotime Biotechnology, China) according to the manufacturer’s instructions. ROS in the NRCMs were detected by a Reactive Oxygen Species Assay Kit (Beyotime Biotechnology, China). Briefly, NRCMs were incubated with 10 μM/L 2′,7′-dichlorodihydrofluorescein diacetate at 37 °C for 20 min. After washing three times, ROS was observed under an Olympus fluorescence microscope.

### Transmission electron microscopy

Transmission electron microscopy was performed as described previously^28^. Images were observed and acquired using a transmission electron microscope (H-7650B; Hitachi Limited).

### Mitochondrial membrane potential (MMP)

MMP of NRCMs were detected by a JC-1 Fluorescence Probe Kit (Beyotime Biotechnology, China) according to the manufacturer’s instruction. Briefly, NRCMs were incubated with 5 µg/mL JC-1 (5,5′, 6,6′-tetrachloro-1,1′,3,3′-tetraethyl-benzimidazole-carbocyanide iodine) at 37 °C for 20 min. The fluorescence was observed and captured using an Olympus fluorescence microscope.

### Statistical analysis

Data are presented as mean ± SEM. For direct comparisons, statistical significance was calculated by unpaired Student’s *t* tests. For multigroup comparisons, analysis of variance followed by post hoc Tukey’s test was performed. The variances between the groups that are being statistically compared were similar. For animal studies, no randomization and blinding were used. A value of *p* < 0.05 was considered statistically significant.

## Supplementary information

Supplementary Figure S1

Supplementary Figure legend
