# Supplementary figures and images for: Autophagy is involved in the protective effect of p21 on LPS-induced cardiac dysfunction
Source: Cell Death Dis. 2020 Jul 21;11(7):554. doi: 10.1038/s41419-020-02765-7 (PMC7374585; doi:10.1038/s41419-020-02765-7)

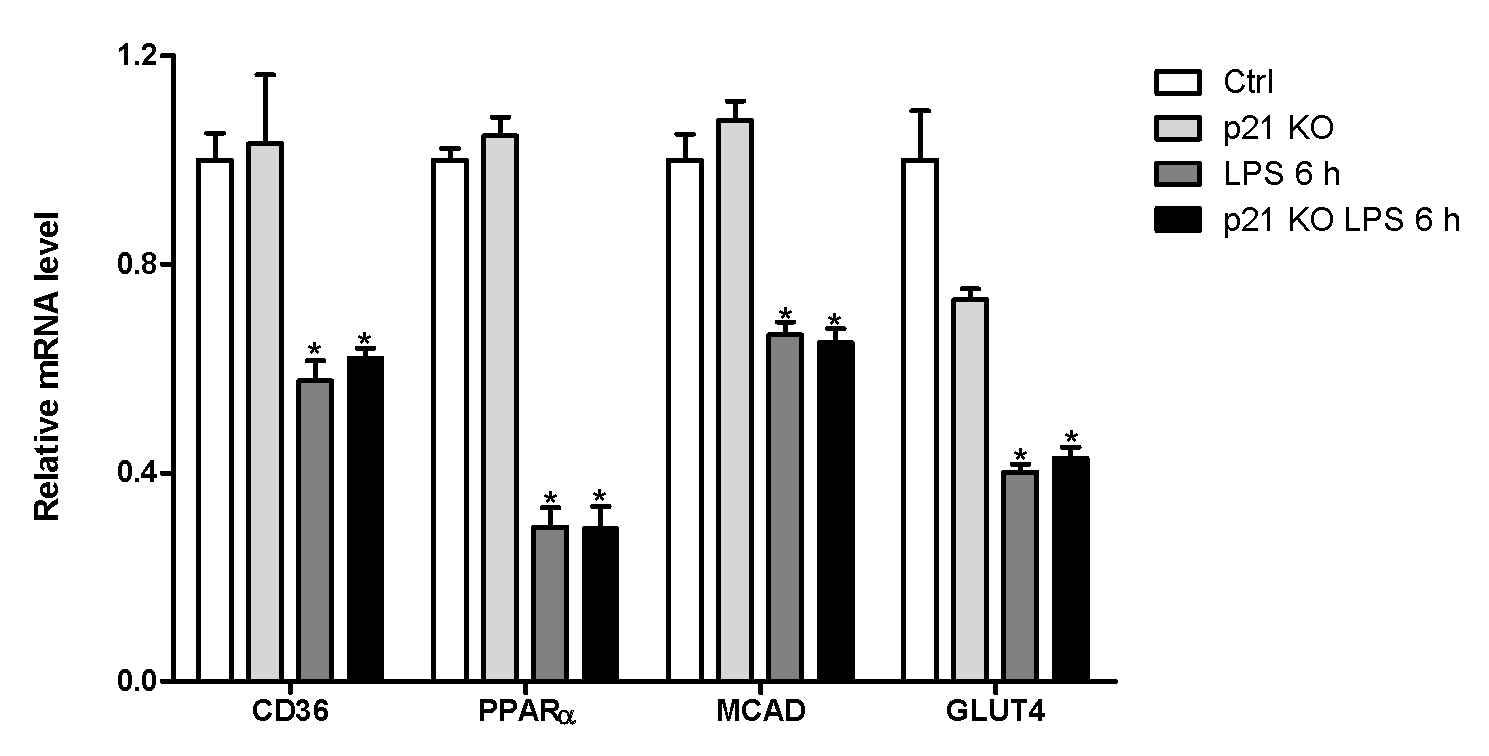

Supplement: Supplementary file 1 — Supplementary Figure S1 [file 41419_2020_2765_MOESM1_ESM.tif]
