# Supplementary material for: Autophagy is involved in the protective effect of p21 on LPS-induced cardiac dysfunction
Source: Cell Death Dis. 2020 Jul 21;11(7):554. doi: 10.1038/s41419-020-02765-7 (PMC7374585; doi:10.1038/s41419-020-02765-7)
Supplement: Supplementary file 2 — Supplementary Figure legend [file 41419_2020_2765_MOESM2_ESM.docx]

**Fig. S1** Relative mRNA level of CD36, PPARα, MCAD and GLUT4 in the WT and p21KO mouse hearts (n=6)
